# Excess Medical Care Costs Associated with Physical Inactivity among Korean Adults: Retrospective Cohort Study

**DOI:** 10.3390/ijerph13010136

**Published:** 2016-01-18

**Authors:** Jin-Young Min, Kyoung-Bok Min

**Affiliations:** 1 Institute of Health and Environment, Seoul National University, Seoul 110-799, Korea; yaemin00@snu.ac.kr; 2 Department of Preventive Medicine, College of Medicine, Seoul National University, 103 Daehak-ro, Jongno-gu, Seoul 110-799, Korea

**Keywords:** exercise, medical cost, burden of disease, chronic disease, propensity score

## Abstract

Physical inactivity is a major risk factor for chronic diseases and premature death. The increased health risks associated with physical inactivity may also generate a heavier economic burden to society. We estimated the direct medical costs attributable to physical inactivity among adultsusing data from the 2002–2010 Korean National Health Insurance Service-National Sample Cohort. A total of 68,556 adults whose reported physical activity status did not change during the study period was included for this study. Propensity scores for inactive adults were used to match 23,645 inactive groups with 23,645 active groups who had similar propensity scores. We compared medical expenditures between the two groups using generalized linear models with a gamma distribution and a log link. Direct medical costs were based on the reimbursement records of all medical facilities from 2005 to 2010. The average total medical costs for inactive individuals were $1110.5, which was estimated to be 11.7% higher than the costs for physically active individuals. With respect to specific diseases, the medical costs of inactive people were significantly higher than those of active people, accounting for approximately 8.7% to 25.3% of the excess burden. Physical inactivity is associated with considerable medical care expenditures *per capita* among Korean adults.

## 1. Introduction

Physical inactivity isa significant public health problem. Despite the beneficial effects of being sufficiently active on both physical fitness and health [1,2,3], more than one-third of the world’s population fail to meet the minimum recommendations for physical activity [4]. The prevalence of inactivity reached 17% in 2009 [5]. The available data suggest that, on a global level, physical inactivity is responsible for 6% to 10% of major non-communicable diseases, including coronary heart disease, certain cancers, and type 2 diabetes [3]. The literature has also indicated that a change from physical inactivity to physical activity could translate into the prevention of 5.3 million of the 57 million deaths attributable to non-communicable diseases and a 0.68 year increase in life expectancy [3].

The increased health risks associated with physical inactivity may also generate a heavier economic burden. Although studies varywith respect to the data and methodologies used, the economicburden of physical inactivity on health care costshas been shown to be substantial [6,7,8,9,10,11]. Inactive people have greater total medical costs than active people. Studies have also demonstrated the excess annual costs caused by the relative risk of physical inactivity for particular diseases [6,7,11]. With the growing recognition of the inactivity-related health care burden beyond the actual effects on health, it is critical to quantify the medical care costs incurred by various diseases associated with physical inactivity and to assess the changes in medical costs over time.

In this study, we examined direct medical costs for physically inactive adults (aged 40–69 years) compared with the costs associated with physically active individuals using the 2002–2010 Korean National Health Insurance Service-National Sample Cohort (KNHIS-NSC). Medical expenditures included total medical costs and specific medical costs resulting from the treatment of specific diseases, for which physical inactivity has been identified as a risk factor, including cardiovascular, endocrine and metabolic, musculoskeletal, respiratory, digestive, neurological, and psychiatric diseases. We estimated the direct medical costs for all disease and for specific diseases for the period 2005–2010.

## 2. Materials and Methods

### 2.1. Data Source and Study Population

Korea's National Health Insurance system is a compulsory social insurance program coveringthe entire population with assistance from government subsidies. The KNHIS-NSC was retrospectivelyobtained from the January 2002 to December 2010cohort database (DB) of the Korean National Health InsuranceCorporation. The dataconstruction procedure is described in detail elsewhere [12,13].

Briefly, the KNHIS-NSC was extracted from the Korean National Health Insurance database. To ensurethat the sample was representative of the Korean population, a stratified random sampling design was used based on characteristics such as age, gender, income, residential area, and annual medical expenses; a total of 1,002,031 people were selected as a random sample of the population. The KNHIS-NSC was composed of a qualification DB, a treatment DB and an examination DB. The qualification DB included basic personal information, such as the resident registration number, residential address, national health insurance-related information, insurance fees, income and family relations. The treatment DB provided detailed annual treatmenthistories, including personal medical records, treatment, procedures, and prescription drugs. The examination DB included resultsfrom healthexaminations conducted through the National HealthInsurance Corporation.

**Figure 1:**
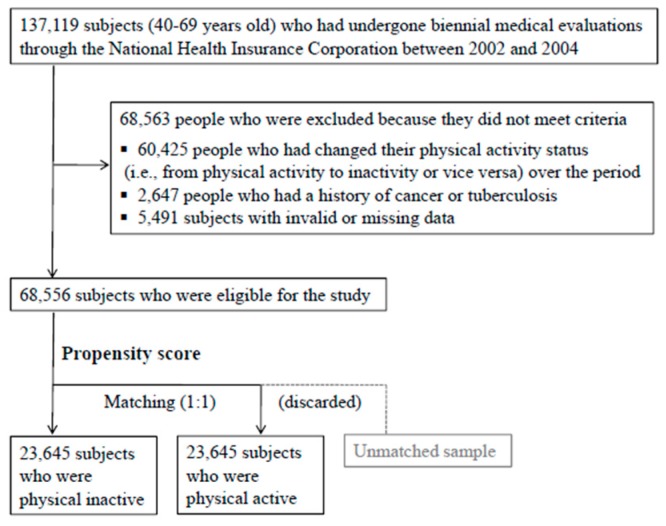
Flow chart of study population selection.

We initially limited the current study to 137,119 subjects (40–69 years old) who had undergone biennial medical evaluations through the National HealthInsurance Corporation between 2002 and 2004. We excluded 60,425 people who had changed their physical activity status (*i.e.*, from physical activity to inactivity or *vice versa*) over the study period. We also excluded 2647 subjects who reported a history of cancer or tuberculosis at the evaluations and 5491 subjects with invalid or missing data; thus, 68,556 subjectsremained in the sample.After estimating propensity score and matching in a one to one ratio, the cohort used in the analysis of adults who were physically active *vs.* physically inactive included 47,290 subjects (Figure 1).

All subjects gave their informed consent for inclusion before they participated in the study. The study was conducted in accordance with the Declaration of Helsinki, and the protocol was approved by the Ethics Committee of Ajou University Hospital (AJIRB-SBR-EXP-14-233). 

### 2.2. Measurements

Participants who underwent a biennial health examination responded to several health-related questionnaires seeking information on health behaviors and disease history. Among these questions, physical inactivity was based on the question “how many times a week do you exercise enough to work up a sweat?” (with responses including none, 1–2 times, 3–4 times, 5–6 times, and almost every day). We categorized the response of none as “physical inactivity” and 1–2 times or more per week as “physical activity”. We defined people who were continuously physically inactive for the study period (2002–2010) as “physically inactivity” and those who were continuously active as “physically active”.

Baseline characteristics were based on the responses collected in 2002–2004 and included age (40–49, 50–59, or 60–69 years), gender (male or female), income (Q1–Q4), residential area (rural or urban), cigarette smoking (yes or no), and alcohol drinking (yes or no). Body mass index (BMI) was calculated based on weight and height measurements obtained during medical evaluations, and the respondents were categorized as normal weight or less (<25.0 kg/m^2^) and overweight or more (≥25.0 kg/m^2^).

Morbidity was based on the claim data, and the specific diseases categories included cardiovascular diseases (ICD: C00–C99), endocrine and metabolic diseases (ICD: E00–E99), musculoskeletal diseases (ICD: M00–M99), respiratory diseases (ICD: J00–J99), digestive diseases (ICD: K00–K99), neurological diseases (ICD: G00–G99), and psychiatric diseases (ICD: F00–F99).

Direct medical costs were calculated as the sum of inpatient, outpatient, and prescription costs based on the reimbursement records of all medical facilities from 2005 to 2010. We converted these costs into the corresponding international dollars (IDs) based on 2012 purchasing powerparity.

### 2.3. Statistical Analysis

To reduce the bias caused by the differences between two groups (physical active group *vs.* physical inactive group), the propensity score-matching method was used for this analysis [14]. Propensity scores for inactive adults were calculated from multivariable logistic regression model including age, gender, income, residential area, cigarette smoking, alcohol consumption, and BMI, and then were used to match 23,645 inactive adults with 23,645 active adults who had similar propensity scores. Unadjusted baseline comparisons between physical inactive and physical active groups were based on the Chi-square tests.

As is typical with medical expenditure data, a large fraction of the samples included individuals who did not receive any care (zero spending) during the period of observation, and the health care expenditures are thus highly skewed.Therefore, we used multivariate generalized linear models with a gamma distribution and a log link to test differences in medical expenditures between physical active groups and inactive groups after adjustments for all baseline characteristics. The models predicted total and specific medical expenditures separately for adults with positive medical care costs. The physically inactive populationwas compared with active adults by calculating the mean and percentage difference in predicted medical costs.All analyses were performed using SAS 9.2 (SAS Institute, Cary, NC, USA), and the level of statistical significance was set at α = 0.05.

## 3. Results

Baseline characteristics of study population are summarized in Table 1. After matching, active and inactive individuals were similar in age, gender, income, residential area, and smoking or alcohol behaviors. There was a significant difference in the BMI distribution between them (*p* < 0.0001).

**Table 1:** Baseline characteristics of the study population: physical active groups and physically inactive groups (2002–2004) unit: *n* (%).

Variables	Active Group		Inactivity Group		*p*-Value *
	(*n* = 23,645)		(*n* = 23,645)		
Age (year)					
40–49	11,791	(50.1)	11,755	(49.9)	0.0737
50–59	7077	(49.3)	7269	(50.7)	
60–69	4777	(50.8)	4621	(49.2)	
Gender					
Female	12,517	(49.9)	12,559	(50.1)	0.6988
Male	11,128	(50.1)	11,086	(49.9)	
Income (won)					
Q1	4576	(51.0)	4403	(49.0)	0.0667
Q2	5450	(49.1)	5649	(50.9)	
Q3	5415	(50.2)	5365	(49.8)	
Q4	8204	(49.9)	8228	(50.1)	
Residential area					
Rural	11,605	(49.7)	11,733	(50.3)	0.2391
Urban	12,040	(50.3)	11,912	(49.7)	
Cigarettesmoking					
No	16,899	(49.8)	17,026	(50.2)	0.1946
Yes	6746	(50.5)	6619	(49.5)	
Alcohol drinking					
No	13,267	(49.7)	13,452	(50.4)	0.0862
Yes	10,378	(50.5)	10,193	(49.6)	
BMI (kg/m^2^)					
Normal or less (BMI < 25)	15,043	(48.6)	15,898	(51.4)	<0.0001
Overweight or more (BMI ≥ 25)	8602	(52.6)	7747	(47.4)	

* *p*-value for the Chi-square test statistic.

**Figure 2:**
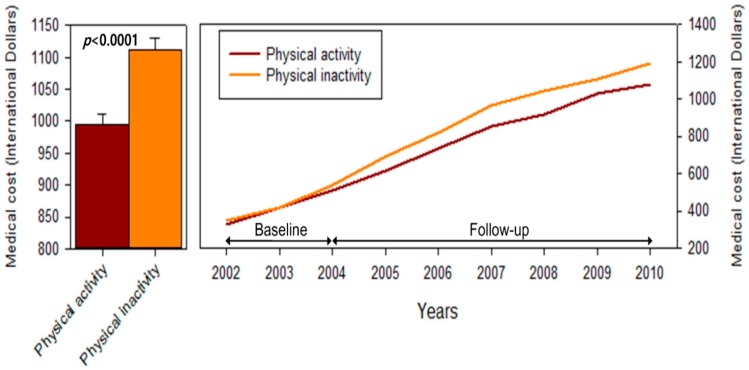
Medical expenditures for physically inactive and active people.

Figure 2 depicts medical expenditures for physically inactive and active people.The left column comparesthe average medical expensesfrom 2005 to 2010 between physical inactive and physical active groups, while the right figure presents the changes in age-adjusted medical expenditures during the study periods between the two groups. The total medical expenditures for physically inactive people were higher than those for active people.

Table 2 reports estimates of the per capita accumulated medical costs for all diseases and for specific diseases among inactive and active people for the 2005 to 2010 period. The estimates were derived from the least-squares regression results among subjects who accrued expenditures. The average total medical cost of inactive individuals was $1110.5, which was estimated to be 11.7% higher than the costs for physically active individuals. With respect to specific diseases, the medicalcostsof inactive peoplewere significantly higher than those of active people, accounting for approximately 8.7% to 25.3% of the excess burden: $902.8 *vs.* $767.8 for cardiovascular diseases, $932.9 *vs.* $744.5 for endocrine and metabolic diseases, $475.6 *vs.* $422.8 for musculoskeletal diseases, $380.1 *vs.* $324.1 for respiratory diseases, $665.1 *vs.* $572.3 for digestive diseases, $723.5 *vs.* $665.4 for neurological diseases, and $572.5 *vs.* $489.9 for psychiatric diseases, respectively.

**Table 2:** Estimates of *per capita* accumulated medical costs: physically active groups and physically inactive groups (2005–2010) unit: international dollars.

Variables	Active Group		Inactivity Group		% Increase ^†^	*p*-Value
	Mean *(95% CI)		Mean *(95% CI)			
Total medical expenditures	994.1	(977.0–1011.6)	1110.5	(1091.0–1130.3)	11.7	<0.0001
Medical costs from specific diseases						
Cardiovascular disease (*n* = 29,263)	767.8	(747.3–788.7)	902.8	(878.2–928.1)	17.6	<0.0001
Endocrine and metabolic diseases (*n* = 28,204)	744.5	(723.9–765.5)	932.9	(906.7–959.9)	25.3	<0.0001
Musculoskeletal disease (*n* = 40,891)	422.8	(414.2–431.6)	475.6	(465.8–485.6)	12.5	<0.0001
Respiratory disease (*n* = 42,778)	324.1	(316.9–331.5)	380.1	(371.3–389.0)	17.3	<0.0001
Digestive disease (*n* = 44,259)	572.3	(560.5–584.2)	665.1	(651.2–679.3)	16.2	<0.0001
Neurologic disease (*n* = 22,057)	665.4	(641.6–690.1)	723.5	(697.6–750.3)	8.7	0.0002
Psychiatric disease (*n* = 20,700)	489.9	(472.3–508.1)	572.5	(551.9–594.0)	16.9	<0.0001

* Mean medical costs predicted from a generalized linear regression model with a gamma distribution and a log link controlling for age, gender, income, residential area, cigarette smoking, alcohol consumption, and BMI; **^†^** Percentage of health care expenditures was calculated by dividing the sum of differences in health care expenditures for inactive adults compared to being active by the total predicted expenditures for all adults.

## 4. Discussion

We found that physical inactivity is associated with considerable per capita medical care expenditures among Korean adults. During the period from 2005 to 2010, physical inactivity generated an excess burden on per capita medical costs, accounting for 11.7% of all diseases and 8.7% to 25.3% of specific diseases including cardiovascular, endocrine and metabolic, musculoskeletal, respiratory, digestive, neurological, and psychiatric diseases. Our findings suggest that the increased health risks associated with physical inactivity may aggravate the healthcare burden.

Physical inactivity is a leading risk factor for non-communicable diseases and premature mortality [3,15]. Relative to active people, physically inactive peopleexhibiteda higher risk of cardiovascular and metabolic disease, obesity, musculoskeletal health problems, and symptoms of anxiety and depression [16,17,18,19]. Overall, studies have demonstrated that there is a substantial economic burden attributable to physical inactivity [6,7,8,9,10,11]. Specifically, Begg *et al.* estimated the burden attributable to physical inactivity as 6.6% of the total burden of disease and 23.7% of the total cardiovascular disease burden in Australia [20]. Martina *et al.* noted that, on an annual basis, 1.4 million cases of diseaseand 1.6 and 0.8 billion Swiss francs in direct and indirect costs, respectively, were attributable to physical inactivity [21]. Allender *et al.* indicated that physical inactivitywas responsible for £1.06 billion of the direct health cost burdenin the UK, of which a large proportion was due to ischmic heart disease (£526 million), stroke (£347 million) and diabetes mellitus (£101 million) [6]. Katzmarzyk *et al.* estimated a considerable public health burden associated with physical inactivity, accounting for 2.6% ($5.3 billion) of the total health care costs in Canada in 2001 [8]. The burden generated from coronary artery disease ($1.7 billion), osteoporosis ($1.5 billion), and stroke ($765 million) represented a large proportion of this total. Garrett *et al.* estimated the total medical expenditures attributable to physical inactivity among adult Blue Cross members in Minnesota [7]. The authors found that $83.6 million ofthe expenditure was attributable to physical inactivity, of particularly related to heart disease ($35.3 million for ischemic heart disease and $10.8 million for hypertension), stroke ($10.8 million), and depression and anxiety ($9.1 million). Notably, although studies differ in the methods used to estimate the burden of disease attributable to physical inactivity, physical inactivity is increasingly imposing a clear and substantial burden on the population and on health care services. Consistent with our findings, physical inactivity imposes substantial medical costs when considered from the perspective of all or specific diseases.

To the best of our knowledge, this work is the first retrospective study to estimate the cumulative or annual medical costs *per capita* attributable to physical inactivity over a 5-year follow-up period while allowing for important comparisons of the medical care burden for all diseases and for specific diseases between physically active and inactive groups. However, several limitations should be considered. The NHID-cohort was based on medical expensesfor which medical institutions submitted reimbursement claimsand includeonly the amount paid by insurance;the DB did not include costs that were not covered or out-of-pocket payments, copayments, and non-national health insurance coverage. This limitation may have led to an underestimation of the direct medical costs associated with physical inactivity. In addition, we used propensity score matching approach to balance the covariates in the two groups but cannot exclude the possibility of bias resulting from residual confounding variable effects or reverse causation related to physical inactivity.

## 5. Conclusions

In conclusion, this study estimated direct medical costs for all disease and for specific diseases—cardiovascular, endocrine and metabolic, musculoskeletal, respiratory, digestive, neurological, and psychiatric diseases-between physically inactive and physically active adults. This study showed that the economic burden of physical inactivity in Korea appears to be substantial. Effective strategies for encouraging physical activity may facilitate numerous health improvements and offer significant health and economic benefits.
